# Transthoracic delivery of large devices into the left ventricle through the right ventricle and interventricular septum: preclinical feasibility

**DOI:** 10.1186/1532-429X-15-10

**Published:** 2013-01-18

**Authors:** Majdi Halabi, Kanishka Ratnayaka, Anthony Z Faranesh, Michael S Hansen, Israel M Barbash, Michael A Eckhaus, Joel R Wilson, Marcus Y Chen, Michael C Slack, Ozgur Kocaturk, William H Schenke, Victor J Wright, Robert J Lederman

**Affiliations:** 1 Cardiovascular and Pulmonary Branch, Division of Intramural Research, NHLBI, NIH, Building 10, Room 2c713, MSC 1538, Bethesda, MD, 20892-1538, USA; 2 Division of Veterinary Resources, NIH, Bethesda, MD, USA; 3 Department of Cardiology, Children’s National Medical Center, Washington, DC, USA

**Keywords:** Catheterization, Interventional cardiovascular MR, Structural heart disease

## Abstract

**Background:**

We aim to deliver large appliances into the left ventricle through the right ventricle and across the interventricular septum. This transthoracic access route exploits immediate recoil of the septum, and lower transmyocardial pressure gradient across the right versus left ventricular free wall. The route may enhance safety and allow subxiphoid rather than intercostal traversal.

**Methods:**

The entire procedure was performed under real-time CMR guidance. An “active” CMR needle crossed the chest, right ventricular free wall, and then the interventricular septum to deliver a guidewire then used to deliver an 18Fr introducer. Afterwards, the right ventricular free wall was closed with a nitinol occluder. Immediate closure and late healing of the unrepaired septum and free wall were assessed by oximetry, angiography, CMR, and necropsy up to four weeks afterwards.

**Results:**

The procedure was successful in 9 of 11 pigs. One failed because of refractory ventricular fibrillation upon needle entry, and the other because of inadequate guidewire support. In all ten attempts, the right ventricular free wall was closed without hemopericardium. There was neither immediate nor late shunt on oximetry, X-ray angiography, or CMR. The interventricular septal tract fibrosed completely. Transventricular trajectories planned on human CT scans suggest comparable intracavitary working space and less acute entry angles than a conventional atrial transseptal approach.

**Conclusion:**

Large closed-chest access ports can be introduced across the right ventricular free wall and interventricular septum into the left ventricle. The septum recoils immediately and heals completely without repair. A nitinol occluder immediately seals the right ventricular wall. The entry angle is more favorable to introduce, for example, prosthetic mitral valves than a conventional atrial transseptal approach.

## Background

Implanting large appliances into the heart, such as investigational transcatheter mitral or aortic valves, will require large introducer sheaths. Transfemoral arterial access may be limited by vessel caliber or disease [1]; transvascular retrograde aortic access risks atheroembolism and stroke [2]. Minithoracotomy access to the left ventricular (LV) apex [3,4] or ascending aorta [5] provides a “straight shot” for large rigid devices, but requires surgical repair. Transcatheter access across the interatrial septum creates unfavorable entry angles and working space [6,7]. A subxiphoid route into the left ventricle would allow delivery of large and rigid implants if introducers could enter and exit gracefully.

We have proposed closed-chest direct transthoracic closure of large access ports directly across the LV free wall using nitinol cardiac closure devices [8], and of direct transthoracic closure of ventricular septal defect across the right ventricular free wall [9]. We noticed while attempting to create an animal model of muscular ventricular septal defect, that aggressive dilatation without ablation of the interventricular septum failed to induce a persistent defect or shunt [9]. Moreover, knife injury across the interventricular septum often fails to induce persistent myocardial defects once hemopericardium is treated [10,11]. Liu and colleagues [12] report a different surgical approach to the LV across the RV free wall and interventricular septum in animals.

These observations led us to propose an alternative closed-chest large-port access route intended (1) to allow entry across the mitral valve orifice; (2) to exploit the reduced leakage pressure gradient across the RV free wall to allow safe closure compared with leakage pressure across the LV free wall; (3) to test the immediate recoil and late healing of large unrepaired sheath ports across the interventricular septum; and (4) to allow a subxiphoid rather than intercostal route.

We hypothesize that 18Fr sheaths can be introduced into beating LV in animals successively across the chest wall, RV free wall, and interventricular septum; that the interventricular septum closes immediately and thereafter fibroses completely; and that the large RV free wall hole can be closed with a nitinol closure device intended to seal atrial septal defect. We perform these procedures in swine using real-time CMR guidance to provide global imaging context of the transthoracic procedure.

## Methods

### Animals

Animal procedures were approved by the institutional animal care and use committee according to contemporary NIH guidelines. The study was performed in 11 naïve Yorkshire pigs (49 ± 6 kg), after a period of technical development in separate animals. Anesthesia was induced with ketamine, midazolam and glycopyrrolate and maintained with inhaled isoflurane and mechanical ventilation. For survival experiments, percutaneous catheters (see below) were removed and animals provided a fentanyl patch and ketoprofen analgesia as needed.

### Catheters

We customized an 18 G CMR-compatible nitinol needle to enhance its visibility during CMR [13]. By adding solenoid receiver coils along the shaft of the needle, we created an antenna that can be connected to the CMR scanner as a receive coil. This “active” antenna detects a signal that can be reconstructed on the CMR scanner on a separate channel. We depict “active” devices in color (in this case, green) overlaid upon the greyscale tissue image, allowing the user to recognize a specific imaging appearance of the device [14].

**Figure 1:**
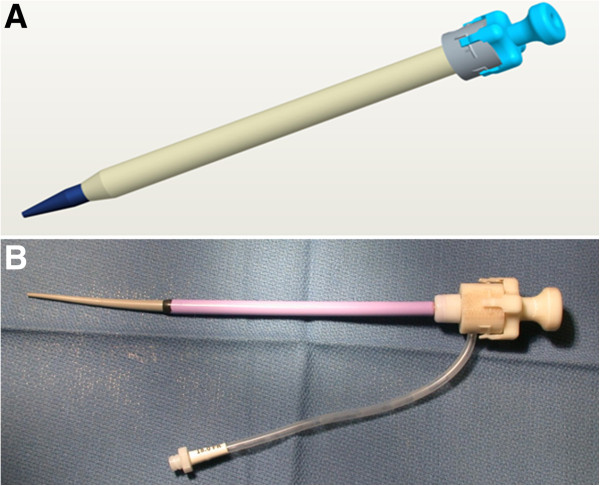
The 18Fr sheath is modified by shortening (to 15 cm) and using a 3D-printed locking dilator (A, blue).

After accessing the LV cavity, off-the-shelf stiff nitinol wires (0.038”Angled Stiff Shaft *Glidewire*, Terumo Medical Corporation or *Nitrex* Guidewire, EV3 Inc.) were used to exchange sequentially for an 8Frx10cm short introducer sheath (*Pinnacle* introducer sheath, Terumo) and then a custom 18Fr introducer sheath. We customized a commercial introducer (Large *Check*-*Flo*, Cook Medical, Bloomington, IN) to shorten the sheath from 30 to 15 cm, and we designed and printed a dilator lock to secure it to the sheath hub (P450 ABS modeling material, *Uprint* rapid prototype printer, Stratsys, Eden Prairie, MN), shown in Figure 1.

At the conclusion of procedures, the RV free wall entry site was closed using an off-the-shelf nitinol atrial septal occluder (*Amplatzer Septal Occluder*, courtesy of St Jude Medical, St. Paul, MN). We modified the delivery cable to incorporate a loopless antenna to enhance visibility during CMR [15].

### CMR

**Figure 2:**
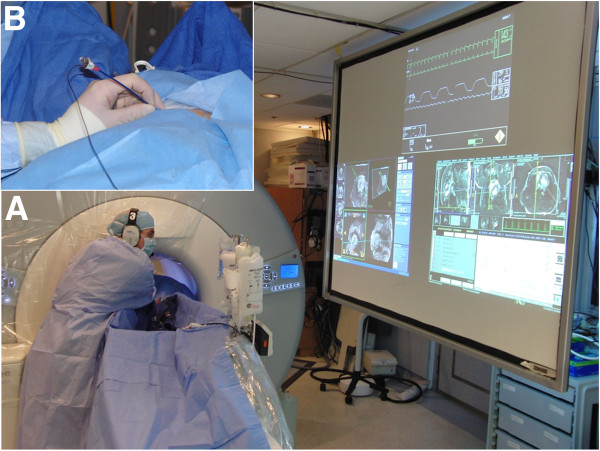
**The interventional CMR environment used here.** Panel (**A**) shows the operator leaning into the 70 cm bore to establish transthoracic access during real-time CMR. LCD projectors display instantaneous hemodynamics, scanner control, and CMR. Acoustic noise suppression headsets allow communication with staff in the control room. Inset (**B**) shows the access port with the table moved out of the scanner, viewed from the left leg.

The CMR suite (1.5 T *Espree* Siemens, Germany) is configured for interventional guidance as described previously [8] , and as shown in Figure 2. The 70 cm scanner bore allows chest access. A commercial prototype workstation (*Interactive Front End*, Siemens) connected to the CMR scanner provides a graphic user interface for interactive slice and scan control and low-latency reconstruction of real-time multislice CMR. The prototype is further customized with NHLBI features including interactive slice thickness, interactive projection mode, and interactive saturation magnetization preparation. In-lab LCD projectors display scanner control, real-time imaging, and instantaneous hemodynamics. Operators wear acoustic noise cancellation headsets and microphones to communicate all at once to team members in- and outside the laboratory (IMROC, Optoacoustics, Moshav Mazor, Israel).

Anatomy and function scans used ECG-gated, segmented breath-hold balanced steady-state free precession (bSSFP) CMR, as well as late gadolinium enhancement using gadopentetate-dimeglumine 0.2 mmol/kg. Typical real-time CMR parameters were: bSSFP; FOV, 350 mm; slice thickness, 6 mm; TR/TE, 2.88/1.44 ms; flip angle 40 degrees, matrix, 192×144, parallel imaging rate 2, temporal resolution 242 ms per slice. Images were constructed and projected on a screen inside the MR suite for the operators.

High resolution *ex*-*vivo* CMR examined the transseptal tract scar. 3-dimensional T1-weighted spoiled gradient echo achieved 0.6 mm isotropic voxel resolution using TR/TE, 10.0/5.35 ms; 8 averages, 100-120slices per slab; flip angle, 20°; matrix, 320×320×144; FOV, 200 mm.

### Interventional procedure

Separate non-survival experiments were performed in 9 animals to explore initial techniques. Early failure modes included: inadequate skin incision to allow large-sheath entry; and inadequate guidewire support to deliver the 18Fr introducer sheath. Thereafter we performed prospective survival experiments on 11 additional animals reported here.

At baseline, a subxiphoid pericardial drain was placed at baseline to instill fluid to separate the pericardial walls and allow appropriate positioning of the epicardial disc of the nitinol closure device (“permissive pericardial tamponade”) [16]. Percutaneous sheaths were placed into the femoral artery and vein. Baseline LV and RV angiography, and pulmonary artery catheterization were performed with oximetry.

CMR guided three key procedure steps. First, a transthoracic needle trajectory was selected. Considerations include avoiding interposed bony structures and intercostal spaces, minimal interposed lung, and a pathway from the RV free wall towards the interventricular septum that enters the mid-LV cavity without papillary muscle injury or entrapment. The trajectory was planned interactively using a finger on the chest surface, and images were stored as a roadmap for subsequent CMR slice prescription. It is important to note that while we are modeling a subxiphoid procedure for human use, the rotation of the porcine heart requires a right lateral transthoracic trajectory in these experiments.

Next the needle was advanced percutaneously during real-time CMR using two interleaved long-axis images for guidance. The shallow needle trajectory is ergonomically suitable for both animal and operator inside the CMR bore. LV cavity entry was confirmed with imaging and pressure measurements before a passive 0.035” × 150 cm nitinol guidewire (Angled Standard Glidewire, Terumo, Somerset, NJ) was used to place a small (8Fr × 10 cm) introducer sheath. Next an air-filled balloon wedge endhole catheter (7Fr, Arrow-Teleflex) was exchanged into the LV and manipulated into a pulmonary vein or the descending aorta to achieve safe guidewire purchase for the next step. Next a stiff 0.038” nitinol guidewire (Angled Stiff *Glidewire*, Terumo) delivered a large 18Fr introducer port modified as described above (Catheter Devices).

No implant was delivered during these experiments, which test access and closure only. The final step is withdrawal of the port and closure of the RV free wall entry site. Because the 18Fr sheath has no discrete imaging characteristics, the balloon wedge endhole catheter was used in tandem with the sheath to depict its tip during withdrawal into the RV. A coaxial 8Frx23cm sheath was used to position the nitinol closure device across the RV free wall. 100–150 mL of saline was injected via the separate pericardial catheter, lowering the blood pressure ~25%, in order to separate the pericardial walls and allow the proximal (epicardial) disc to be deployed along the epicardial border.

### Follow-up

Follow-up was designed to detect early or late hemopericardium, iatrogenic myocardial dysfunction, and to characterize the extent of myocardial injury caused by the trans-ventricular access tract.

After RV closure the animals were observed for two hours, before follow-up CMR and catheter angiographic and oximetric assessment.

Late CMR follow-up was performed on day 4 and week 4, including cine CMR for function and pericardial effusion, and late gadolinium enhancement for interventricular septum inspection. Animals were euthanized after four weeks and the heart extracted for high resolution *ex*-*vivo* late gadolinium enhanced (3D T1-weighted) CMR. Necropsy and histopathology by a dedicated veterinary pathologist evaluated the presence of ventricular septal defect or scar.

### Trajectory planning in humans

We assessed the anatomic feasibility of transthoracic interventricular septal access in humans using an anonymized and de-linked series of adults with suspected cardiovascular disease undergoing contrast-enhanced computed tomography (CT, 320×0.5-mm detector rows, Aquillion One, Toshiba Medical Systems, Japan) extending to the abdomen using ECG-gated breath-held acquisitions (courtesy of M.Y. Chen, NHLBI DIR). These do not constitute human subject research under US 45CFR§46.102(f).

### Data analysis

LV mass, function, scar volume, and trajectory geometries were measured using a CMR workstation (Leonardo, Siemens). Paired continuous parameters were compared using a Student t-test. Serial continuous parameters were tested using ANOVA. A p value < 0.05 was considered significant.

## Results

### Feasibility of direct RV-septal- LV access under real-time CMR guidance

After initial technique development in 9 animals, survival experiments were performed in 11 additional swine, and were successful in 9. One failed because of refractory ventricular fibrillation immediately after LV puncture. In the other, the large sheath failed to deliver because we had not yet learned to use deep guidewire support in the left atrium or aorta. This animal recovered uneventfully after RV port closure. The technique was modified in response to both (oral and intravenous amiodarone loading, and deep guidewire support after needle entry).

**Figure 3:**
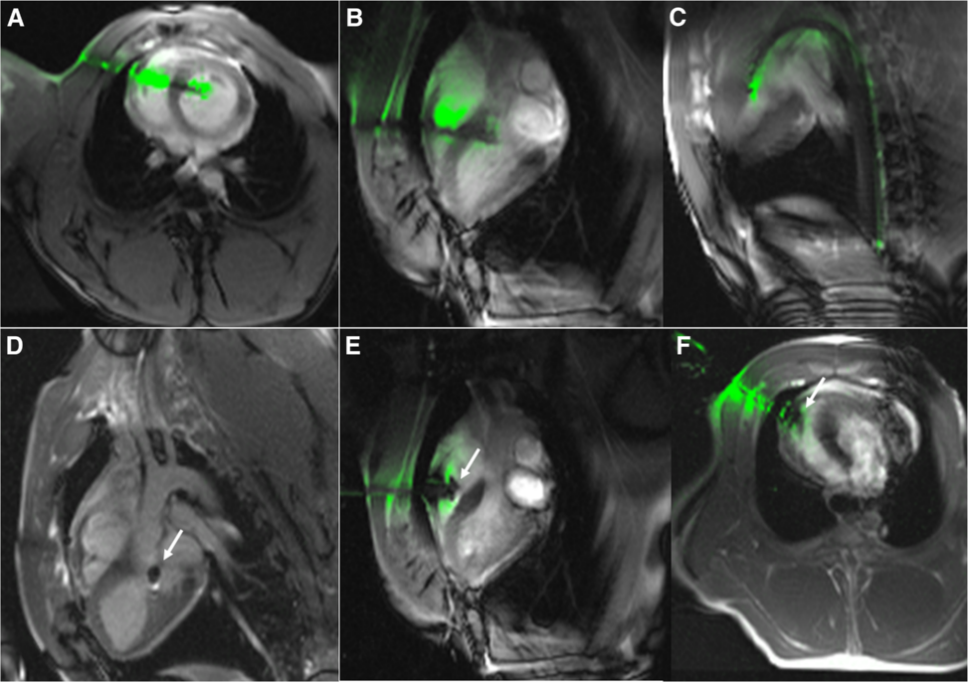
**Real-time CMR of a typical procedure.** (**A,B**) Simultaneous views of needle across chest and septum into the LV. (**C**) Guidewire through needle into descending aorta. (**D**) Balloon catheter indicates sheath tip (arrow) as it is withdrawn. (**E, F**) RV free wall closure using a nitinol occluder (arrow).

Figure 3 depicts a typical procedure sequence. The active needle, attached to a separate CMR receiver channel, is depicted in green during real-time CMR. In this animal, the guidewire positioned in the descending aorta couples electrically with the active needle and therefore also appears in green. After the 18Fr port is introduced into the LV across the interventricular septum, its tip is visualized using an air-filled balloon catheter (panel 1D) as it is repositioned into the RV. Finally the RV entry hole is sealed with a nitinol atrial septal occluder device. No therapeutic procedure is performed in this experiment.

No ventricular septal defect was evident immediately after port closure, either by oximetry (Table 1), X-ray angiography, or by CMR at 0 or 2 hours.

**Table 1:** Hemodynamics and oximetry

	**Baseline**	**2 hours post procedure**	**p value**
**Hemodynamics**			
Heart rate beats/min	81 ± 18	97 ± 28	0.17
Mean arterial pressure mmHg	54 ± 10	51 ± 6	0.50
**Oxygen Saturation**			
Femoral artery%	95 ± 0.6	95 ± 0.7	0.20
Right atrium%	71 ± 6.2	66 ± 7.5	0.20
RV%	72 ± 6.7	67 ± 7.8	0.16
Pulmonary artery%	72 ± 7.1	66 ± 8.8	0.14
Qp/Qs	0.89 ± 0.01	0.93 ± 0.02	0.80
Hemoglobin (g/dL)	8.4 ± 3.9	7.0 ± 0.7	0.30

### RV free wall closure

After the 18Fr (7.3 mm sheath outer diameter) port was introduced across the RV into the LV, the RV free wall hole was closed under real-time CMR guidance using an Atrial Septal Occluder 6-8 mm (St Jude Medical, Figure 3, panels E-F).

When we downsized the RV sheath from 18Fr to 8Fr to deploy the nitinol occluder, pericardial fluid leaked around the access site and failed to accumulate in 3/9 cases. As a result, the epicardial disc of the nitinol occluder failed to seat inside the pericardial space. Nevertheless, RV free wall closure was successful in 9/9 attempts, and without hemopericardium.

### Evaluation of the interventricular septal tract and of the myocardium

Baseline ejection fraction as measured by CMR was 55 ± 6% and remained unchanged after 2 hours, 4 days and 4 weeks (p = 0.70), including among individual animals. No regional wall motion abnormality was detected.

**Figure 4:**
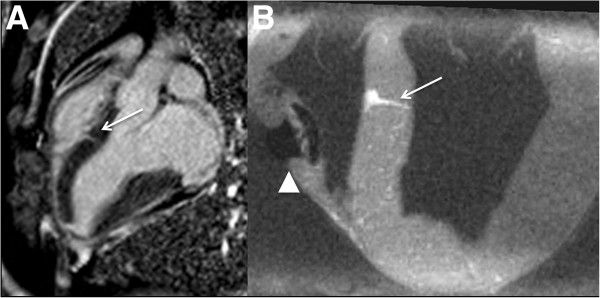
***In-vivo *** **(A) and high-resolution *ex-vivo *** **late-gadolinium CMR of the healed interventricular septum tract (arrow) and nitinol occluder (arrowhead) after four weeks.**

**Figure 5:**
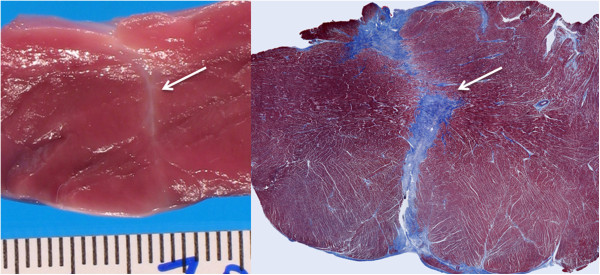
Fresh macroscopic and Masson trichrome (collagen-avid) microscopic images of the interventricular septal tract (arrow) after four weeks, showing fibrosis and no evident bystander injury.

**Figure 6:**
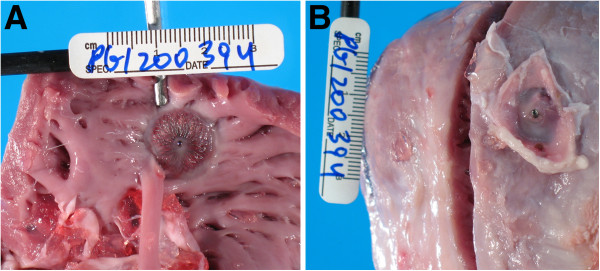
**Macroscopic specimen of the ASD device from the RV cavity (A) and outside the RV free wall (B) four weeks after implantation.** The endocardial surface is partially fibrosed.

Late gadolinium enhancement CMR *in*-*vivo* revealed a low-volume interventricular septal scar along the sheath tract. This tract was better depicted using high-resolution *ex*-*vivo* CMR (Figure 4). The calculated scar volume was 0.16 ± 0.03 g (0.18% ± 0.02 of the LV mass). Histopathology after four weeks showed a conical tract 5.1 ± 1.3 mm at the RV side and 3.8 ± 0.75 mm at the LV side that traversed the interventricular septum (Figure 5), with organized fibrosis. There was no evidence of ventricular septal defect. The right ventricular free wall closure device was completely endothelialized on endocardial and epicardial surfaces (Figure 6).

### Complications

There were two cases of ventricular fibrillation during LV puncture or wire passage. In one, resuscitation was unsuccessful (described above), and in the other, after defibrillation the procedure continued without incident.

One animal had a large and two others small reactive (serous) pericardial effusion at 4 weeks, and one had a small right pleural effusion.

There were no cases of hemopericardium after RV free wall closure. There was no evident ECG conduction delay in any animal. There was no evident pneumothorax or lung injury.

### Trajectory planning in humans

**Figure 7:**
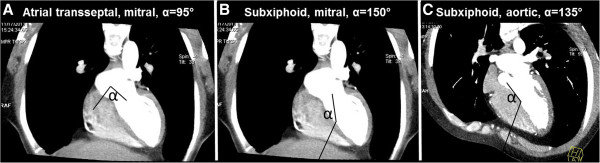
**Theoretical trajectories from human CTA.** (**A**) “Conventional” atrial transseptal trajectory to the mitral valve has entry angle (α) = 95°. (**B**) Subxiphoid trajectory to the mitral valve across the RV and interventricular septum, α = 150°. (**C**) Subxiphoid trajectory to the aortic valve across the RV and interventricular septum, α = 130°. These also depict the distances reported in Table 2.

Although these porcine experiments necessarily used a right lateral transthoracic trajectory, we examined the feasibility of a subxiphoid trajectory to the LV through the RV and interventricular septum in 21 patients (13 men and 8 women; mean age 52 ± 6 years) who underwent cardiac computed tomography with abdominal and thoracic coverage for cardiovascular disease. We measured geometry of theoretical trajectories using our novel subxiphoid-transventricular approach, and using “conventional” approach across the interatrial septum, comparing intracameral “working space” (distance) and catheter angulation (Figure 7).

The distance from the skin to the RV epicardium was 50 ± 12 mm. The distance from the RV epicardium to the LV endocardium through the interventricular septum was 45 ± 6 mm. Table 2 indicates that both transventricular and atrial transseptal approaches for transcatheter mitral valve implantation provided similar intracameral working distance between cavity entry and the target valve annulus, however our transventricular approach afforded a less acute angle (148 ± 6° vs 98.9 ± 5°, p < 0.01).

**Table 2:** Geometry of transventricular and conventional trajectories based on human CT data

	**Transventricular LV endocardial entry across interventricular septum**		**Conventional LA entry through fossa ovalis**	
	**Distance (mm)**	**Angle**	**Distance (mm)**	**Angle**
To aortic valve annulus	43 ± 4	125 ± 6°	-	-
To mitral valve annulus	44 ± 4	148 ± 6° *	37.8 ± 5.7	98.9 ± 5

Distances are reported between chamber entry site (LV endocardial border for transventricular access, LA border for atrial transseptal access) and the center of the target valve annulus. Angles are reported between chamber entry site and a line directly to the center of the target valve annulus. These are depicted graphically in Figure 6. * = p < 0.01 compared with an atrial transseptal trajectory.

## Discussion

We have demonstrated a new closed-chest transthoracic access procedure into the beating LV across the interventricular septum. This approach sequentially traverses the RV free wall and then the interventricular septum instead of the LV free wall. The cavity-to-pericardial (“leakage”) pressure gradient is lower for a RV free wall entry compared with LV free wall entry, and in this way our trajectory may enhance acute procedural safety of closed-chest direct cardiac access. Translated into patients, our approach would allow subxiphoid introduction of large appliances into the heart without surgery, for example transcatheter mitral valves having delivery systems 10 mm wide or more [17]. In this preclinical experience, we found that the entry tract across the interventricular septum recoiled completely, was associated with no early or late ventricular septal defect, was associated with no evident myocardial dysfunction or conduction abnormality, and left a small residual fibrotic scar with no evident bystander injury. A commercial nitinol occluder device successfully closed the RV free wall access port. Real-time CMR guidance enabled straightforward performance of this procedure by providing broad thoracic context and clear soft tissue trajectories. Our review of 21 adult human CT data sets suggests a subxiphoid transcatheter trajectory should be feasible with entry angles that are suitable for treating the aortic and mitral valves, and reduced angulation towards the mitral valve compared with a conventional atrial transseptal approach. We believe this work establishes proof-of-concept for a new closed-chest non-surgical access port procedure to implant large cardiac appliances when transvascular access is not suitable.

To our surprise during the conception of this experiment, the 7.3 mm diameter iatrogenic ventricular septal defect recoiled acutely and then healed spontaneously and completely in every case. This is consistent with reports of spontaneous closure of traumatic ventricular septal defects, summarized by Dehgani and colleagues [11]. This also is consistent with the difficulty we experienced attempting to create an animal model of muscular ventricular septal defect without mural laser ablation [9]. Presumably the immediate recoil facilitates rapid healing, despite the large transseptal pressure gradient, manifest as a clinically-insignificant fibrotic tract after four weeks. The volume of septal scar is comparatively smaller than small CMR defects generated during otherwise uncomplicated percutaneous coronary intervention [18]. Liu and colleagues [12] report a similar “transventricular-transseptal” approach with a 5 cm subxiphoid incision to deliver a transcatheter aortic valve implant. They chose to close the iatrogenic ventricular septal defect with a nitinol occluder where our experience suggests this may be unnecessary.

Possible advantages of our approach compared with surgical or percutaneous transapical LV access include (1) subxiphoid access without intercostal injury and pain for transthoracic transapical access; (2) reduced cameral-pericardial pressure gradient contributing to bleeding across the access tract; (3) lower likelihood of major epicardial coronary artery injury from access across the RV free wall compared with the LV apex; (4) an extrapleural and subxiphoid trajectory reducing the risk of pulmonary injury and avoiding intercostal width and trajectory constraints. Possible disadvantages of our approach include (1) injury to the interventricular septum causing a persistent ventricular septal defect or conduction abnormality, even though we observed neither in this preclinical experience; (2) shorter intracavitary “working distance” to the mitral or aortic valves compared with transapical access, and angulated entry to the aortic valve; (3) possible interference with the endocardial closure device by RV mural trabeculation, a phenomenon we did not observe in this preclinical experience; and (4).pulmonary hypertension related valve disease might reduce the proposed benefit of lower pressure gradient across the RV free wall.

There is little tolerance for risk in a transcatheter alternative to surgery. For this procedure, we have developed techniques to mitigate risk. First, a separate pericardial catheter drain is placed at the beginning of the procedure, intended to separate the pericardial walls by transient fluid installation. This drain can decompress the pericardium in an emergency. Second, an ancillary “buddy” guidewire may allow transcatheter rescue should the nitinol closure device accidentally be withdrawn through the right ventricular free wall. In our prior preclinical report of direct transthoracic repair of ventricular septal defect [9], inadvertent RV pull-through was an observed adverse event for which we proposed to apply a balloon-tipped delivery sheath. However, in the current series, there were no inadvertent RV pull-through events. Third, bailout surgical repair of RV free wall perforation is technically feasible. Finally, preliminary feasibility of device closure of cardiac free walls has been suggested by numerous clinical reports of closure of cardiac perforations using unmodified Amplatzer Septal Occluder devices [19], of transapical LV access ports up to 12Fr using Amplatzer Duct Occluders [20], or of intraoperative closure of perforations with Amplatzer Muscular VSD Occluder [21].

Real-time CMR provided superb guidance for trajectory planning, needle access, guidewire and sheath delivery, and the RV free wall closure. CMR has an advantage over X-ray or ultrasound guidance in providing soft-tissue images in any arbitrary orientation, independent of acoustic windows imposed by ribs or air. Most important, the large field of view provided by real-time CMR provides a global thoracic context of the procedure allowing the operator to avoid bystander tissue injury and monitor instantaneously for complications [8,16].

### Limitations

This work has noteworthy limitations. The procedure succeeded in only 9 of 11 animals. One failure is attributed to needle-induced ventricular fibrillation, which is common in swine. Another failure is attributed to immature catheter technique, from failure to obtain deep purchase of a rigid guidewire in order to deliver the cardiac sheath. Late complications were evident in one animal, manifest as serous (reactive) pericardial effusion without signs of tamponade. In clinical practice we would expect to leave a pericardial drainage catheter in place for one or more days in an attempt to prevent pericardial effusion. Real-time CMR remains investigational, although early human CMR catheterization procedures are being undertaken [22,23].

## Conclusions

We have developed a novel non-surgical approach to introduce large structural implants into the LV. A subxiphoid introducer sheath is placed across the RV free wall and from there across the interventricular septum into the LV. The entry angle for mitral valve implants is less acute than a conventional atrial transseptal trajectory. The RV free wall hole is closed with a nitinol occluder device. Surprisingly, the interventricular septum hole closes immediately and completely, and afterwards heals as a small fibrous tract.

## Abbreviations

ECG: Electrocardiogram; FOV: Field of view; CMR: Cardiovascular magnetic resonance; LV: Left ventricle; RV: Right ventricle; TE: Echo time; TR: Repetition time.

## Competing interests

NIH and Siemens Medical Systems have a collaborative research and development agreement for CMR.

Kanishka Ratnayaka serves without compensation on a Siemens Pediatric Advisory Council. Michael Slack is compensated as physician proctor for St Jude Medical.

## Authors’ contributions

MH designed and performed experiments, analyzed the data, and prepared and edited the manuscript. KR, AZF, MSH, IMB JRW, MYC, MCS, OK, WHS, VJW assisted in experiments and edited the manuscript. MAE performed necropsy and histopathology analysis. RJL designed experiments, analyzed the data, and prepared and edited the manuscript. All authors read and approved the final manuscript.
